# Thermophilic and halophilic β-agarase from a halophilic archaeon *Halococcus* sp. 197A

**DOI:** 10.1007/s00792-013-0575-z

**Published:** 2013-08-15

**Authors:** Hiroaki Minegishi, Yasuhiro Shimane, Akinobu Echigo, Yukari Ohta, Yuji Hatada, Masahiro Kamekura, Tadashi Maruyama, Ron Usami

**Affiliations:** 1 Bio-Nano Electronics Research Center, Toyo University, 2100 Kujirai, Kawagoe, Saitama 350-8585 Japan; 2 Japan Agency for Marine-Earth Science and Technology (JAMSTEC), 2-15, Natsushima-cho, Yokosuka, Kanagawa 237-0061 Japan; 3 Halophiles Research Institute, 677-1, Shimizu, Noda, Chiba, 278-0043 Japan

**Keywords:** *Halococcus*, Thermophilic, Halophilic, β-agarase

## Abstract

An agar-degrading archaeon *Halococcus* sp. 197A was isolated from a solar salt sample. The agarase was purified by hydrophobic column chromatography using a column of TOYOPEARL Phenyl-650 M. The molecular mass of the purified enzyme, designated as Aga-HC, was ~55 kDa on both SDS-PAGE and gel-filtration chromatography. Aga-HC released degradation products in the order of neoagarohexose, neoagarotetraose and small quantity of neoagarobiose, indicating that Aga-HC was a β-type agarase. Aga-HC showed a salt requirement for both stability and activity, being active from 0.3 M NaCl, with maximal activity at 3.5 M NaCl. KCl supported similar activities as NaCl up to 3.5 M, and LiCl up to 2.5 M. These monovalent salts could not be substituted by 3.5 M divalent cations, CaCl_2_ or MgCl_2_. The optimal pH was 6.0. Aga-HC was thermophilic, with optimum temperature of 70 °C. Aga-HC retained approximately 90 % of the initial activity after incubation for 1 hour at 65–80 °C, and retained 50 % activity after 1 hour at 95 °C. In the presence of additional 10 mM CaCl_2_, approximately 17 % remaining activity was detected after 30 min at 100 °C. This is the first report on agarase purified from Archaea.

**Electronic supplementary material:**

The online version of this article (doi:10.1007/s00792-013-0575-z) contains supplementary material, which is available to authorized users.

## Introduction

Agar, the main cell wall component of red macroalgae, is widely used as a gelling agent of microbiological culture media as well as molecular sieving in DNA electrophoresis and gel-filtration chromatography. Agar produced commercially from the species of genera *Gelidium* and *Gracilaria* is composed of agarose and agaropectin (Aoki et al. 1990; Rochas et al. 1986). Agarose consists of 3, 6-anhydro-l-galactose and d-galactose alternately linked by α-1, 3 and β-1, 4 linkages (Kloareg and Quatrano 1988). Agaropectin has the same basic disaccharide-repeating units as agarase with some hydroxyl groups of 3, 6-anhydro-l-galactose residues replaced by sulfoxy or methoxy and pyruvate residues (Hamer et al. 1977).

Agarases are hydrolytic enzymes that degrade agarose into oligosaccharides. They were characterized as either α-agarase (E.C. 3.2.1.158) that cleaves α-1, 3 linkage to produce a series of agaro-oligosaccharides related to agarobiose (Potin et al. 1993) or β-agarase (E.C. 3.2.1.81) that cleaves β-1, 4 linkage to produce neoagarooligosaccharides of series related to neoagarobiose (Kirimura et al. 1999). Agarose-degrading enzymes have been attracting keen interest in many fields of biochemistry and enzymology (Fu and Kim 2010). The neoagarooligosaccharides have various special biological activities, such as inhibition of bacterial growth, slowing down of starch degradation thereby reducing the calorific value of food, and providing anticancer, antivirus, and anti-oxidation activities (Giordano et al. 2006). The β-agarases are also used to recover DNA from agarose gel after electrophoresis (Finkelstein and Rownd 1978; Burmeister and Lehrach 1989).

There have been numerous reports on agarase from isolates belonging to genera of the domain Bacteria, including *Acinetobacter*, *Agarivorans*, *Alteromonas*, *Bacillus*, *Cytophaga*, *Microbulbifer*, *Pseudoalteromonas*, *Pseudomonas*, *Salegentibacter*, *Thalassomonas*, *Vibrio*, *Zobellia*, etc. (Hu et al. 2009; Fu and Kim 2010), many of which are of marine origin. There have been no reports, however, on agarases from halophilic Bacterial strains requiring high (more than 10 %) NaCl concentrations for growth. On the other hand, no reports have been published on agarases from any strains of the domain *Archaea*, although glycoside hydrolases such as α-amylases (Pérez-Pomares et al. 2003; Hutcheon et al. 2005), chitinase (Zhang et al. 2010), cyclodextrin glycosyltransferase (Bautista et al. 2012), β-xylanase and β-xylosidase (Wainø and Ingvorsen 2003), have been identified from halophilic Archaeal strains, as well as from hyperthermophilic Archaea (Kim and Ishikawa 2010; Rashid et al. 2002; Tanaka et al. 2001).

In the present study, we isolated agarase-producing halophilic archaeal strains belonging to the genus *Halococcus* from solar salt samples. We describe purification and characterization of the halophilic and thermophilic β-agarase.

## Materials and methods

### Isolation of agar-degrading extreme halophiles

Agar-degrading extreme halophiles were screened on agar plates of modified JCM medium No. 169, which contained (in per liter) 250.0 g NaCl, 0.75 g casamino acids, 1.0 g yeast extract, 0.3 g trisodium citrate, 2.0 g KCl, 20.0 g MgSO_4_·7H_2_O, 0.05 g FeSO_4_ 4H_2_O, 0.2 g MnSO_4_ 4H_2_O, 20.0 g Bacto-agar (Difco), pH adjusted to 7.2 with 40 % KOH. Approximately 300 salt samples, domestic and imported, were dissolved in 4 ml of 5 % sterile NaCl solution, spread on the agar plates, and incubated at 37 °C for 2 weeks. Two colonies that formed depression or clearing zones on agar were picked up and purified further by the same plating method.

### Tentative identification of isolates

Total DNA was extracted by the method of Cline et al. (1989). The 16S rRNA gene was amplified by PCR with the following forward primer H16S For (5′-CCCTGCGSTCCGSCGT-3′) and reverse primer 23S Rev2 (5′-GCTTATCGCAGCTTGG-3′) (Minegishi et al. 2012). The amplification with Ex Taq DNA polymerase (TaKaRa, Japan) was started by incubation at 96 °C for 5 min, followed by 25 cycles of 20 s at 98 °C, 30 s at 58 °C, and 1 min 30 s at 72 °C, and final extension was done for 2 min at 72 °C. The amplified DNA was cloned by the TA Cloning Kit (Invitrogen) and sequenced using the BigDye Terminator v3.1 Cycle Sequencing Kits (Applied Biosystems) with the following primers; H16S_86F: 5′-GCTCAGTAACACGTGGCCAA-3′ and H16S_351R: 5′-GTAAAGGTTTCGCGCCTGCT-3′ and H16S_314F: 5′-CCGGGCCCTACGGGGCGCAG-3′ and H16S_982F: 5′-GAGAGGAGGTGCATGGCCGC-3′ and H16S_1295R: 5′-CTACCGAATCCAGCTTCATG-3′ on the ABI PRISM 310 Genetic Analyzer (Applied Biosystems).

### Enzyme assay

Agarase activity was determined by measuring the increase of reducing sugar released from agarose using the modified dinitro salicyclic acid (DNS) method (Miller 1959). Unless indicated, Agarose-ME (Medium Electroendosmosis, Nacalai Tesque, Kyoto, Japan), with gelling temperature of 35–40 °C, was used as the substrate. The standard assay condition was as follows: reaction mixture containing 0.9 ml of 0.33 % (w/v) Agarose-ME solution in 3.5 M NaCl, 50 mM 2-Morpholinoethanesulfonic acid (MES)-NaOH buffer (pH 6.0) and 0.1 ml of diluted enzyme solution was incubated at 65 °C for 15 min. The reaction mixture was mixed with 1.0 ml of DNS reagent and boiled for 5 min. The mixture was diluted with 4.0 ml of distilled deionized water, and absorbance was read at 535 nm. For the blanks, we placed reaction mixtures containing 0.1 ml of 2.5 M NaCl, 5 mM Tris–HCl buffer without the enzyme. In some experiments, agarose preparations from other suppliers were also used; Agarose-LE (Nacarai Tesque, Japan), Agarose-S (Nippon Gene, Japan), Certified Molecular Biology Agarose (BIO-RAD), Bacto-agar (Difco), and Agar noble (Difco).

### Production and purification of the agarase

The agarase-producing *Halococcus* sp. was cultivated at 37 °C with rotary shaking at 120 rpm in 2.0 liters of a medium HA (in per liter): 250.0 g NaCl, 2.0 g KCl, 20.0 g MgSO_4_ 7H_2_O, 5.0 g yeast extract, 5.0 g casamino acids, 3.0 g trisodium citrate, 1.0 g sodium glutamate, 36.0 mg FeSO_4_ 4H_2_O, 0.36 mg MnCl_2_ 4H_2_O, pH adjusted to 7.2 with 40 % KOH. The cells at late logarithmic phase were collected aseptically by centrifugation at 8000×*g* for 15 min at 15 °C, then the cell pellet was seeded into 4.0 liters of an induction medium HA-i composed of salt and trace metal components of HA and 0.2 % agarose (Certified Molecular Biology Agarose, Bio-Rad). After incubation for 2–3 days at 37 °C with shaking at 120 rpm, supernatant of the culture obtained by centrifugation was saturated with NaCl, and subjected to hydrophobic column chromatography using a column (2.5 × 15 cm) of TOYOPEARL Phenyl-650 M (TOSOH, Japan) equilibrated with 30 % NaCl, 5 mM Tris–HCl, pH 7.0. After washing with the same buffer, elution was done with the Tris–HCl buffer of step-wise decreasing NaCl concentrations. Fractions with agarase activities were pooled, NaCl added to saturation, loaded to another column of TOYOPEARL Phenyl-650 M (1 × 10 cm), and eluted with a linear decreasing NaCl concentration, from 5.0 M to zero. Agarase-active fractions were collected and dialyzed against 2.5 M NaCl, 5 mM Tris–HCl, pH 7.0, and concentrated with an ultrafiltration device, Amicon Ultra 3 kDa (Millipore). All procedures were done at 4 °C and the relative protein content was estimated by absorbance at 280 nm. Protein concentration of purified enzyme was determined by Lowry method, using bovine serum albumin as the standard.

### Characterization of agarase activity

The effect of salt concentrations on the enzyme activity was determined using 0.3 % agarose in the standard buffer (50 mM MES, pH 6.0) containing 0–5.0 M NaCl, 0–3.5 M KCl, or 0–3.5 M LiCl. Divalent cation salts, 0–3.5 M MgCl_2_ or 0–3.5 M CaCl_2_ at intervals of 0.5 M.

The optimum pH for the enzyme was examined in the following 50 mM buffers with various pH values: citric acid-NaOH (pH 4.0–6.0), MES-NaOH (pH 5.5–7.0), sodium phosphate (pH 6.5–8.0), glycylglycine-NaOH (pH 7.0–8.5), *N*-tris (Hydroxymethyl) methyl-4-aminobutanesulfonic acid-NaOH (TABS-NaOH) (pH 8.5–9.5) under the standard assay condition. The effect of temperature was examined under the standard assay condition except that the temperature was varied from 35 to 100 °C.

Thermal stability of the enzyme (~0.04 mg/ml) was determined in the presence of 3.5 M NaCl by incubation at 65, 70, 80, 90 and 95 (in glass test tubes using thermostated water bath) and 100 °C (in 0.2 ml microtubes using heat block of GeneAmp PCR System 9700, Applied Biosystems) for 0–60 min and the residual activities were measured under the standard assay condition. In a separate experiment, stabilities at 100 °C were measured in the absence or presence of 10 mM CaCl_2_. The enzymatic characterizations were performed three or four times with *n* = 3 for each samples.

### Analysis of hydrolysates of agarose

Agarose-ME was hydrolysed with Aga-HC in the presence of 3.5 M NaCl, 5 mM Tris–HCl, pH 6.0, for 1, 2, 4, 8, and 16 h at 65 °C. The hydrolysates were desalted using ion-exchange resins from Amberlite MB-3 (Organo, Tokyo, Japan) and analyzed by thin-layer chromatography (TLC) on Silica 60 TLC plates (Merck) using chloroform: methanol: acetic acid (3:3:1, v/v) as a developing solvent. Products were visualized by spraying with 10 % (v/v) H_2_SO_4_ and baking at 180 °C. d-galactose, and two oligomers, neoagarotetraose and neoagarohexose prepared according to Ohta et al. (2005), were used as standards. The control experiments were done with α-agarase from *Thalassomonas* *agarivorans* JAMB-A33 (Ohta et al. 2005; Miyazaki et al. 2008) and β-agarase from *Microbulbifer thermotolerans* JAMB-A94 (Ohta et al. 2004; Miyazaki et al. 2008) by hydrolyzing Agarose-ME in 20 mM Tris–HCl buffer for 2, 4, 8, and 16 h at 40 °C.

### Molecular mass determination

Gel-filtration chromatography was done using ÄKTAexplorer 10S System (GE Healthcare Japan) equipped with HiPrep 16/60 Sephacryl S-200 HR column (1.6 × 60 cm, GE Healthcare Japan) with a solvent (1.5 ml/min) of 3.5 M NaCl, 5 mM Tris–HCl, pH 7.0. Ribonuclease A (14.8 kDa), ovalbumin (43.5 kDa), bovine serum albumin (61.3 KDa), aldolase (176 kDa), catalase (219 kDa) were used to determine the apparent molecular weight of an agarase.

Sodium dodecyl sulfate polyacrylamide gel electrophoresis (SDS-PAGE) was performed in a SuperSep 5–20 % polyacrylamide gradient gel (Wako Pure Chemical Industries, Ltd., Japan) with a buffer containing 25 mM Tris, 192 mM glycine, and 0.1 % SDS (pH 8.8). Proteins were stained with Bio-Safe Coomassie stain (BIO-RAD). Precision Plus dual color standards (BIO-RAD, 10–250 kDa) were run as molecular mass standards.

## Results

### Agarase-producing strains

A huge number of colonies appeared on agar plates from ~300 salt samples. We obtained, however, only two strains that formed depression on agar plates, and they also gave halo after flooding with Lugol’s solution (0.2 I_2_ plus 2 % KI). Both strains were red pigmented, coccoid forming microscopically, and did not lyse in distilled water. The 16S rRNA gene sequence (AB748563) of the higher agarolytic strain was closest to those of *Halococcus* *salifodinae* DSM 8989^T^ (AB004877) and *Halococcus* *saccharolyticus* ATCC 49257^T^ (AB004876) with 99.4 and 99.2 % similarity, respectively (Fig. S1). We designated the strain as *Halococcus* sp. 197A and the agarase from this strain as Aga-HC. *Halococcus* sp. 197A showed a depression with a diameter of 20 mm around a colony (about 4 mm) on agar medium after incubation at 37 °C for 2 weeks (Fig. S2). The strain was able to grow up to 45 °C, and grew optimally at around 37 °C with maximum agarase production on agar plates. The two most closely related strains *Hcc.* *salifodinae* JCM 9578^T^ and *Hcc*. *saccharolyticus* JCM 8878^T^ did not show any agarose-degrading activities in our experiments.

### Preparation of Aga-HC

**Fig. 1:**
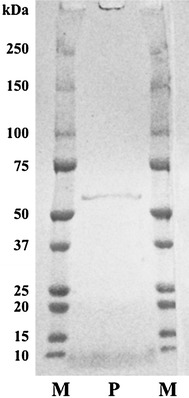
SDS-PAGE of the purified Aga-HC in 5–20 % gradient polyacrylamide gel (*lane P*). Protein mass markers (in kDa) are indicated on the both side (*lane M*). The proteins were stained with Coomassie *brilliant blue*

In liquid culture media, essentially no agarolytic activities were detected. Therefore, we induced the enzyme production in the HA-i medium with 0.2 % agarose as described in Materials and methods. The purified Aga-HC (~0.4 mg/ml) was homogeneous as judged from SDS-PAGE (Fig. 1). Molecular weight was estimated as 56 kDa by SDS-PAGE and 55 kDa by gel-filtration chromatography using ÄKTAexplorer 10S System. The purified Aga-HC was stable for months as long as kept in a refrigerator in 2–3 M NaCl at neutral pH.

### Effects of salt and pH on agarase activity

**Fig. 2:**
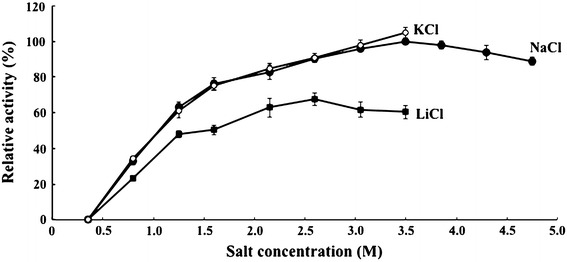
The effect of NaCl, KCl and LiCl concentrations on the activity of Aga-HC determined at 65 °C using 0.3 % agarose in the standard buffer (50 mM MES, pH 6.0) containing 0–5.0 M NaCl and 0–3.5 M of LiCl or KCl at intervals of 0.5 M

**Fig. 3:**
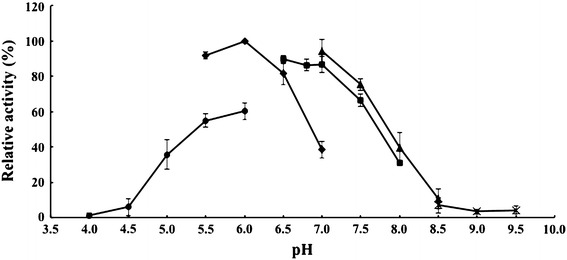
The effect of pH on the activities of Aga-HC at 65 °C in the presence of 3.5 M NaCl. The following 50 mM buffers were used. Citric acid-NaOH (*filled circle*: pH 4.0–6.0), MES-NaOH (*filled diamond*: pH 5.5–7.0), sodium phosphate (*filled square*: pH 6.5–8.0), glycylglycine-NaOH (*filled triangle*: pH 7.0–8.5), *N*-tris (Hydroxymethyl) methyl-4-aminobutanesulfonic acid-NaOH (×: pH 8.5–9.5)

The Aga-HC showed no activity in the presence of 0–0.3 M salt. Increasing activities were observed with the increase of NaCl concentration, with optimum at 3.5 M. In a separate experiment, KCl supported similar activities as NaCl up to 3.5 M, and LiCl to a lesser extent up to 2.5 M (Fig. 2). When these monovalent salts in the reaction mixtures were replaced by divalent cation salts, CaCl_2_ or MgCl_2_, no activity was detected at 0–0.1 M. At higher concentrations, up to 3.5 M, white precipitate [probably Ca(OH)_2_ or Mg(OH)_2_] appeared upon addition of DNS reagent, which was strongly alkaline. Absorbances (at 535 nm) of supernatants after boiling and spinning down did not increase, suggesting these divalent cations did not support activities of the agarase. The agarolytic activity was inactivated when dialysed against a NaCl-free buffer for 24 h, and recovery of activity was not observed after re-dialysis against 3.5 M NaCl, 5 mM Tris–HCl, pH 7.0 for 3 days. The enzyme showed activities from pH 4.5 to 8.5, with optimum at around pH 6 (Fig. 3).

### Effects of temperature on activity and stability

**Fig. 4:**
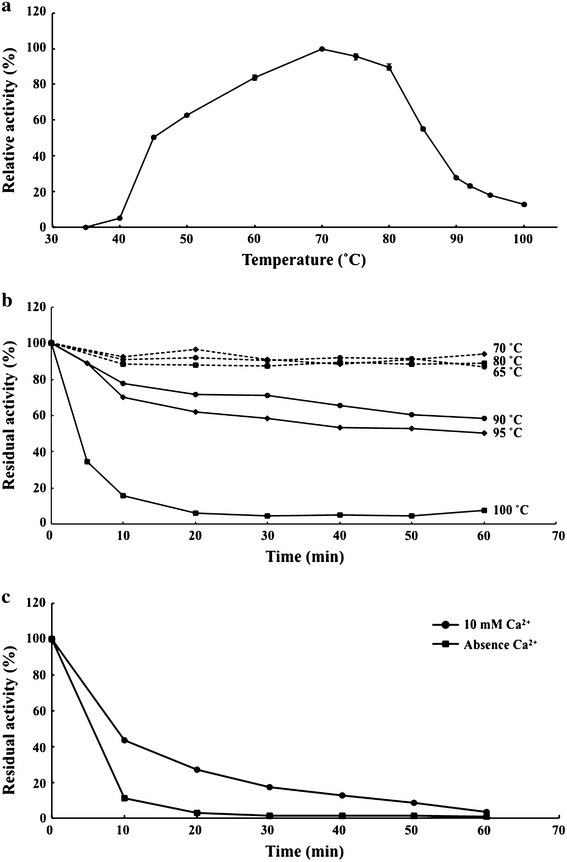
Effect of temperature on the enzyme activity and stability of purified Aga-HC. The enzyme activity was measured at temperatures ranging from 35 to 100 °C using MES-NaOH buffer (pH 6.0) (**a**). For the thermal stability of Aga-HC, the enzyme was incubated at indicated temperatures for 0–60 min in the absence (**b**) or presence of 10 mM CaCl_2_ (**c**), and remaining activities were measured at 65 °C as described in “Materials and methods”

In our standard assay condition (reaction for 15 min with final agarose-ME concentration of 0.27 %), Aga-HC showed no activity at temperature lower than 35 °C, because of formation of soft gel. Increasing agarolytic activities were observed with the increase of temperature from 40 °C up to 70 °C, then decreasing activities were obtained until 100 °C (Fig. 4a). Non-enzymatic degradation of agarose was not detected. Measurement of stability of the enzyme (in 3.5 M NaCl, 5 mM Tris–HCl, pH 7.0) at high temperatures revealed its high thermostability. Aga-HC retained ~90 % of the initial activities after incubation for 1 hour at 65–80 °C (Fig. 4b). When incubated at 95 °C, more than 50 % activity remained, while almost no activity was detected after incubation at 100 °C for 30 min. In the presence of additional 10 mM CaCl_2_, ~17 % remaining activity was detected after 30 min at 100 °C (Fig. 4c).

### Substrate specificity and the reaction products

The enzyme showed degradation activity on agar or agarose from various suppliers as determined by the release of reducing sugar. Relative activities were expressed with that against Agarose-ME as 100 %; 97 % to Agarose-LE, 69 % to Agarose-S, 70 % to Agarose, 77 % to Bacto-agar, and 72 % to Agar noble.

**Fig. 5:**
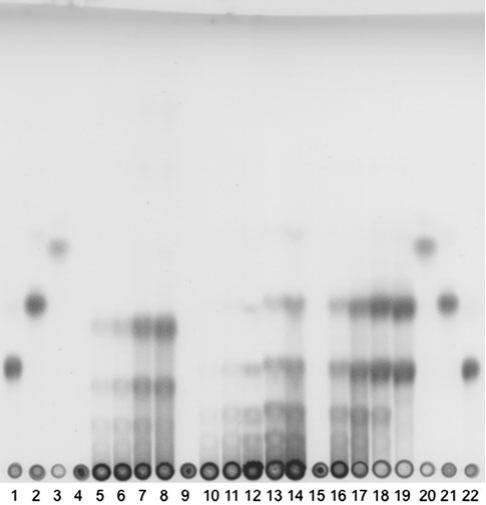
Thin-layer chromatogram of hydrolysis products by three agarases on Silica 60 TLC plates (Merck).* Lanes* 4–8, α-agarase from *Thalassomonas* *agarivorans* JAMB-A33 (after incubation at 40 °C for 0, 2, 4, 8, 16 h), *lanes* 9–14, Aga-HC (after incubation at 65 °C for 0, 1, 2, 4, 8, 16 h), *lanes* 15–19, β-agarase from *Microbulbifer thermotolerans* JAMB-A94 (after 0, 2, 4, 8, 16 h at 40 °C). Markers were neoagarohexaose (*lanes* 1 and 22), neoagarotetraose (*lanes* 2 and 21), and galactose (*lanes* 3 and 20). A developing solvent was chloroform: methanol: acetic acid (3:3:1, v/v) and products were visualized by spraying with 10 % (v/v) H_2_SO_4_ and baking at 180 °C

Oligosaccharides in a hydrolysate of Agarose-ME at 65 °C were analyzed by TLC after desalting of the reaction mixture as described in materials and methods. Aga-HC released degradation products in the order of neoagarohexose, neoagarotetraose and small quantity of neoagarobiose (Fig. 5). The spot with slightly higher Rf value than galactose of lane 14 (after incubation for 16 h) was neoagarobiose (Ohta et al. 2005). These data indicated that Aga-HC was a β-type agarase.

## Discussion

According to Stanier (1941), the first isolation of an agar-digesting bacterium was reported by HH Gran in 1902, while engaged in a general study of marine bacteria. Decomposition of agar in ocean is of undoubted importance in the cycle of matter in the ocean, where agar and similar polysaccharides form a large part of the carbohydrate constituents of many marine algae. As summarized by Fu and Kim (2010) many of agar-digesting Bacteria are of marine origin. Then, what about organisms thriving in solar saltern, a hypersaline extreme environment of seawater origin? Although numerous halophilic Archaea have been isolated from salterns and salt lakes throughout the world (Oren 2007; Enache et al. 2012), there have been no description on agar-degrading activities so far in more than 140 species belonging to *Halobacteriaceae.* We also have not encountered any noticeable depression of agar around colonies during our exploration for extreme halophiles possessing new phenotypic characteristics. In this study, we paid special attention to depression of more than 300 agar plates, and luckily enough we succeeded in isolating two agar-degrading strains from two solar salt samples.

At present, genome sequences of 145 Archaeal strains have been made public (Kyoto Encyclopedia of Genes and Genomes), and three strains have been suggested to possess agarase genes, *Thermosphaera aggregans* in the order *Desulfurococcales*, *Thermococcus sibiricus* in the order *Thermococcales* and *Halalkalicoccus jeotgali* in the order *Halobacteriales*. The agarase of *T. aggregans* and *T. sibiricus* have not been characterized yet (Spring et al. 2010; Mardanov et al. 2009). The haloarchaeal strain *H. jeotgali* JCM 14584^T^ was reported to have been isolated from a Korean seafood produced with sea salt (Roh et al. 2007). This strain, however, showed no depression on the agar plate in our experiments. Thus, this is the first report on agarase from the domain *Archaea*.

The purified Aga-HC from our Archaeal strain *Halococcus* sp. 197A was halophilic and lost activity in the absence of NaCl as do most enzymes from halophilic Archaea (Madern et al. 2000). On the other hand, agarases from Bacteria are known to require low NaCl concentrations for their optimum activities as is the case of most other enzymes, for example 0.15 M for β-agarase II from *Pseudomonas atlantica* (Morrice et al. 1983) and 0.9 M for β-agarase PjaA from *Pseudomonas* sp. strain W7 (Ha et al. 1997). The β-agarase (Thermostable β-Agarase; Wako Pure Chemical Industries, Ltd., Japan) from *Microbulbifer* *thermotolerans* showed almost the same activities up to 1.25 M NaCl, and decreasing activities were observed with increasing NaCl concentrations. (Fig. S3a).

Most agarases of bacterial strains work optimally below 40 °C (Fu and Kim 2010). The β-agarase from *Microbulbifer* *thermotolerans* JAMB-A94 of marine origin was exceptional in that the optimum temperature was 55 °C (Ohta et al. 2004). Moderately thermophilic bacteria, *Alterococcus agarolyticus*, growing at 38–58 °C isolated from hot springs were reported to produce extracellular agarase on agar medium (Shieh and Jean 1998), but further studies on the strain and agarase have not been published. In our assay condition, Aga-HC exhibited no detectable activity at temperature below 35 °C since the 0.3 % agarose formed a soft gel below this temperature. Papers dealing with agarases working at low temperature have coped with this difficulty using much lower agarose concentration, e.g. 0.005 % (Morrice et al. 1983). The Aga-HC was extraordinary in showing high activities at temperature higher than 40 °C and it worked optimally at as high as 70 °C in the presence of 3.5 M NaCl. It has long been suggested that some, not all, enzymes from halophilic archaeal strains were thermophilic. In a now classical paper by Keradjopoulos and Holldorf (1977), they stated that ‘salt lakes and brines are often closed and stable hydrologic systems, in which intensive isolation and a reduced circulation cause temperatures up to 70 °C in layers of high salinity near the surface’. They demonstrated that nine enzymes of three strains of *Halobacterium salinarum* exhibited maxima of temperature for catalytic activities between 55 and 70 °C; those enzymes were thermophilic. Since then, a number of papers have dealt with halophilic and thermophilic enzymes from haloarachaea. For example, a serine protease from a haloarchaeal strain 172P1 (now *Natrialba asiatica*) exhibited high activity at 75–80 °C, when assayed in the presence of 25 % NaCl. The optimal concentration of NaCl required was 10–14 % when assayed at 70° (Kamekura and Seno 1990). A β-xylanase of a halophilic archaeon *Halorhabdus utahensis* exhibited optimal activity at 70 °C (Wainø and Ingvorsen 2003). More recently, recombinant alcohol dehydrogenase from the haloalkaliphilic archaeon *Natronomonas pharaonis* was shown to be most active at 5 M NaCl or 4 M KCl and 70 °C (Cao et al. 2008).

Aga-HC was also extraordinary in that it was quite thermostable. In the presence of 3.5 M NaCl, pH 7.0, the enzyme retained approximately 90 % of the initial activity after incubation for 1 hour at as high as 80 °C. Most agarases of bacterial strains are stable below 50 °C (Fu and Kim 2010). For example, the β-agarase from *Microbulbifer* *thermotolerans* JAMB-A94 was stable at 50 °C for 15 min in 20 mM Tris–HCl buffer (pH 7.0), while retained only 10 % activity after incubation at 70 °C for 15 min (Ohta et al. 2004). Our separate experiments showed that the enzyme was thermolabile in the presence of 2.5 M NaCl (Fig. S3b).

Likewise, β-agarases from *Alteromonas* sp. SY37-12 (Wang et al. 2006), *Agarivorans albus* YKW-34 (Fu et al. 2008), and *Acinetobacter* sp. Ag LSL-1 (Lakshmikanth et al. 2009) retained activities after incubation as follows; 20 % after 1 min at 70 °C, 10 % after 60 min at 70 °C, and none after 60 min at 60 °C, respectively.

The *Halococcus* sp. 197A that produced Aga-HC exhibiting the highest halophilicity (3.4 M NaCl for optimum), thermophilicity (70 °C for optimum) and thermostability (half-life of 60 min at 95 °C) in agarases reported so far was isolated from a solar salt sample. This fact raises some interesting and enigmatic questions; where is the indigenous habitat of the strain, what is the enzyme doing, and where did the gene of Aga-HC come from? Some haloarchaeal strains have been shown to survive in sea water (Inoue et al. 2011) and in 0.5 % NaCl solution for as long as 10 days (Fukushima et al. 2007). *Halococcus* species were found in the nostrils salt glands of the seabird *Calonectris diomedea* (Brito-Echeverría et al. 2009). Due to the restricted flow of seawater into Hamelin Pool, Shark Bay, Australia and the high net evaporation rates, the salinity of the surface water in Hamelin Pool is twice that of normal seawater and the living stromatolites are partially submerged in this hypersaline environment. *Halococcus hamelinensis* (Goh et al. 2006; Burns et al. 2012) was isolated from a stromatolite in Hamelin pool. Although *Hcc. hamelinensis* strain 106A6 is not an agarase producer, agarase-producing *Halococcus* strains or strains of other genera might be thriving in saline environments throughout the world. More extensive survey for agarase-producing halophiles might give some clues to the decomposition of agar in saline environments as well as in the ocean.

## Electronic supplementary material

Below is the link to the electronic supplementary material.

Phylogenetic tree of *Halococcus* sp. 197A and species of the genus *Halococcus* based on 16S rRNA gene sequences using neighbor-joining method. The bootstrap values were generated from 1,000 replicates (TIFF 1521 kb)

Depression of an agar plate and clearance zone after staining with Lugol’s solution (0.2 % I_2_ plus 2 % KI). A drop of cell suspension of *Halococcus* sp. 197A was placed on the center of an agar plate and incubated at 37 °C for 2 weeks (TIFF 1521 kb)

The effect of NaCl on the enzyme activity and temperature stability of Thermostable β-Agarase from *Microbulbifer* *thermotolerans*. The enzyme activity was measured at NaCl concentrations ranging from 0.25 to 4.75 M using 5 mM Tris–HCl buffer (pH 7.0) (**a**) (TIFF 1521 kb)

For the thermal stability, the enzyme was incubated at 50 °C for 0–60 min in the absence (*filled circles*) or presence of 2.5 M NaCl (*filled square*), and remaining activities were measured at 50 °C for 15 min (TIFF 1521 kb)

## References

[CR1] Aoki T, Araki T, Kitamikado M (1990). Purification and characterization of a novel beta-agarase from *Vibrio* sp. AP-2. Eur J Biochem.

[CR2] Bautista V, Esclapez J, Pérez-Pomares F, Martínez-Espinosa RM, Camacho M, Bonete MJ (2012). Cyclodextrin glycosyltransferase: a key enzyme in the assimilation of starch by the halophilic archaeon *Haloferax mediterranei*. Extremophiles.

[CR3] Brito-Echeverría J, López-López A, Yarza P, Antón J, Rosselló-Móra R (2009). Occurrence of *Halococcus* spp. in the nostrils salt glands of the seabird *Calonectris diomedea*. Extremophiles.

[CR4] Burmeister M, Lehrach H (1989). Isolation of large DNA fragments from agarose gels using agarase. Tren Genet.

[CR5] Burns BP, Gudhka RK, Neilan BA (2012). Genome sequence of the halophilic archaeon *Halococcus hamelinensis*. J Bacteriol.

[CR6] Cao Y, Liao L, Xu XW, Oren A, Wang C, Zhu XF, Wu M (2008). Characterization of alcohol dehydrogenase from the haloalkaliphilic archaeon *Natronomonas pharaonis*. Extremophiles.

[CR7] Cline SW, Schalkwyk LC, Doolittle WF (1989). Transformation of the archaebacterium *Halobacterium volcanii* with genomic DNA. J Bacteriol.

[CR8] Enache M, Popescu G, Itoh T, Kamekura M, Stan-Lotter H, Fendrihan S (2012). Halophilic microorganisms from man-made and natural hypersaline environments: physiology, ecology, and biotechnological potential. Adaptation of microbial life to environmental extremes.

[CR9] Finkelstein M, Rownd TH (1978). A rapid method for extracting DNA from agarose gels. Plasmid.

[CR10] Fu XT, Kim SM (2010). Agarase: review of major sources, categories, purification method, enzyme characteristics and applications. Mar Drugs.

[CR11] Fu XT, Lin H, Kim SM (2008). Purification and characterization of a novel beta-agarase, AgaA34, from *Agarivorans albus* YKW-34. Appl Microbiol Biotechnol.

[CR12] Fukushima T, Usami R, Kamekura M (2007). A traditional Japanese-style salt field is a niche for haloarchaeal strains that can survive in 0.5% salt solution. Sal Sys.

[CR13] Giordano A, Andreotti G, Tramice A, Trincone A (2006). Marine glycosyl hydrolases in the hydrolysis and synthesis of oligosaccharides. Biotechnol J.

[CR14] Goh F, Leuko S, Allen MA, Bowman JP, Kamekura M, Neilan BA, Burns BP (2006). *Halococcus hamelinensis* sp. nov., a novel halophilic archaeon isolated from stromatolites in Shark Bay, Australia. Int J Syst Evol Microbiol.

[CR15] Ha JC, Kim GT, Kim SK, Oh TK, Yu JH, Kong IS (1997). β-agarase from *Pseudomonas* sp. W7: purification of the recombinant enzyme from *Escherichia coli* and the effects of salt on its activity. Biotechnol Appl Biochem.

[CR16] Hamer GK, Bhattacharjee SS, Yaphe W (1977). Analysis of the enzymic hydrolysis products of agarose by ^13^C-n.m.r spectroscopy. Carbohydr Res.

[CR17] Hu Z, Lin BK, Xu Y, Zhong MQ, Liu GM (2009). Production and purification of agarase from a marine agarolytic bacterium *Agarivorans* sp. HZ105. J Appl Microbiol.

[CR18] Hutcheon GW, Vasisht N, Bolhuis A (2005). Characterisation of a highly stable α-amylase from the halophilic archaeon *Haloarcula hispanica*. Extremophiles.

[CR19] Inoue K, Itoh T, Ohkuma M, Kogure K (2011). *Halomarina oriensis* gen. nov., sp. nov., a halophilic archaeon isolated from a seawater aquarium. Int J Syst Evol Microbiol.

[CR20] Kamekura M, Seno Y (1990). A halophilic extracellular protease from a halophilic archaebacterium strain 172 P1. Biochem Cell Biol.

[CR21] Keradjopoulos D, Holldorf AW (1977). Thermophilic character of enzymes from extreme halophilic bacteria. FEMS Microbiol Lett.

[CR22] Kim H-W, Ishikawa K (2010). Complete saccharification of cellulose at high temperature using endo cellulase and β-glucosidase from *Pyrococcus* sp. J Microbiol Biotechnol.

[CR23] Kirimura K, Masuda N, Iwasaki Y, Nakagawa H, Kobayashi R, Usami S (1999). Purification and characterization of a novel β-agarase from an alkalophilic bacterium, *Alteromonas* sp. E-1. J Biosci Bioeng.

[CR24] Kloareg B, Quatrano RS (1988). Structure of the cell walls of marine algae and ecophysiological functions of the matrix polysaccharides. Oceanogr Mar Biol Annu Rev.

[CR25] Lakshmikanth M, Manohar S, Lalitha J (2009). Purification and characterization of β-agarase from agar-liquefying soil bacterium, *Acinetobacter* sp., AG LSL-1. Proc Biochem.

[CR26] Madern D, Ebel C, Zaccai G (2000). Halophilic adaptation of enzymes. Extremophiles.

[CR27] Mardanov AV, Ravin NV, Svetlitchnyi VA, Beletsky AV, Miroshnichenko ML, Bonch-Osmolovskaya EA, Skryabin KG (2009). Metabolic versatility and indigenous origin of the archaeon *Thermococcus sibiricus*, isolated from a siberian oil reservoir, as revealed by genome analysis. Appl Environ Microbiol.

[CR28] Miller L (1959). Use of dinitrosalicylic acid reagent for determination of reducing sugar. Anal Chem.

[CR29] Minegishi H, Kamekura M, Kitajima-Ihara T, Nakasone K, Echigo A, Shimane Y, Usami R, Itoh T, Ihara K (2012). Gene orders in the upstream of 16S rRNA genes divide genera of the family *Halobacteriaceae* into two groups. Int J Syst Evol Microbiol.

[CR30] Miyazaki M, Nogi Y, Ohta Y, Hatada Y, Fujiwara Y, Ito S, Horikoshi K (2008). *Microbulbifer agarilyticus* sp. nov. and *Microbulbifer thermotolerans* sp. nov., agar degrading bacteria isolated from deep-sea sediment. Int J Sys Evol Microbiol.

[CR31] Morrice LM, McLean MW, Williamson FB, Long WF (1983). β-agarases I and II from *Pseudomonas**atlantica*. Purifications and some properties. Eur J Biochem.

[CR32] Ohta Y, Nogi Y, Miyazaki M, Li Z, Hatada Y, Ito S, Horikoshi K (2004). Enzymatic properties and nucleotide and amino acid sequences of a thermostable β-agarase from the novel marine isolate, JAMB-A94. Biosci Biotechnol Biochem.

[CR33] Ohta Y, Hatada Y, Miyazaki M, Nogi Y, Ito S, Horikoshi K (2005). Purification and characterization of a novel α-agarase from a *Thalassomonas* sp. Curr Microbiol.

[CR34] Oren A, Gerday C, Glansdorff N (2007). Biodiversity in highly saline environments. Physiology and biochemistry of extremophiles.

[CR35] Pérez-Pomares F, Bautista V, Ferrer J, Pire C, Marhuenda-Egea FC, Bonete MJ (2003). Alpha-amylase activity from the halophilic archaeon *Haloferax mediterranei*. Extremophiles.

[CR36] Potin P, Richard C, Rochas C, Kloareg B (1993). Purification and characterization of the α-agarase from *Alteromonas agarlyticus* (Cataldi) comb. nov., strain GJ1B. Eur J Biochem.

[CR37] Rashid N, Cornista J, Ezaki S, Fukui T, Atomi H, Imanaka T (2002). Characterization of an archaeal cyclodextrin glucanotransferase with a novel C-terminal domain. J Bacteriol.

[CR38] Rochas C, Lahaye M, Yaphe W, Phanviet MT (1986). ^13^C-N.M.R.-spectroscopic investigation of agarose oligomers. Carbohydr Res.

[CR39] Roh SW, Nam YD, Chang HW, Sung Y, Kim KH, Oh HM, Bae JW (2007). *Halalkalicoccus jeotgali* sp. nov., a halophilic archaeon from shrimp jeotgal, a traditional Korean fermented seafood. Int J Sys Evol Microbiol.

[CR40] Shieh WY, Jean WD (1998). *Alterococcus agarolyticus*, gen. nov., sp.nov., a halophilic thermophilic bacterium capable of agar degradation. Can J Microbiol.

[CR41] Spring S, Rachel R, Lapidus A, Davenport K, Tice H, Copeland A, Cheng JF, Lucas S, Chen F, Nolan M, Bruce D, Goodwin L, Pitluck S, Ivanova N, Mavromatis K, Ovchinnikova G, Pati A, Chen A, Palaniappan K, Land M, Hauser L, Chang YJ, Jeffries CC, Brettin T, Detter JC, Tapia R, Han C, Heimerl T, Weikl F, Brambilla E, Göker M, Bristow J, Eisen JA, Markowitz V, Hugenholtz P, Kyrpides NC, Klenk HP (2010). Complete genome sequence of *Thermosphaera aggregans* type strain (M11TL). Stand Genom Sci.

[CR42] Stanier RY (1941). Studies on marine agar-digesting bacteria. J Bacteriol.

[CR43] Tanaka T, Fukui T, Imanaka T (2001). Different cleavage specificities of the dual catalytic domains in chitinase from the hyperthermophilic archaeon *Thermococcus kodakaraensis* KOD1. J Biol Chem.

[CR44] Wainø M, Ingvorsen K (2003). Production of β-xylanase and β-xylosidase by the extremely halophilic archaeon *Halorhabdus utahensis*. Extremophiles.

[CR45] Wang J, Mou H, Jiang X, Guan H (2006). Characterization of a novel β-agarase from marine *Alteromonas* sp. SY37-12 and its degrading products. Appl Microbiol Biotechnol.

[CR46] Zhang Y, An R, Yatsunami R, Sato M, Orishimo K, Hatori Y, Fukui T, Nakamura S (2010). Characterization of a haloarchaeal GH family 18 chitinase with additional acidic amino acids on its protein surface. J Jpn Soc Extremophiles.

