# Supplementary figures and images for: Thermophilic and halophilic β-agarase from a halophilic archaeon Halococcus sp. 197A
Source: Extremophiles. 2013 Aug 15;17(6):931–9. doi: 10.1007/s00792-013-0575-z (PMC3824881; doi:10.1007/s00792-013-0575-z)

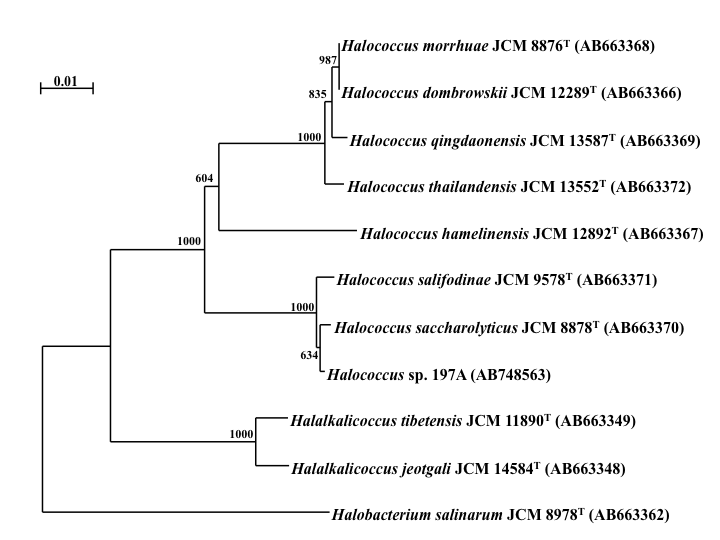

Supplement: Supplementary file 1 — Phylogenetic tree of Halococcus sp. 197A and species of the genus Halococcus based on 16S rRNA gene sequences using neighbor-joining method. The bootstrap values were generated from 1,000 replicates (TIFF 1521 kb) [file 792_2013_575_MOESM1_ESM.tiff]

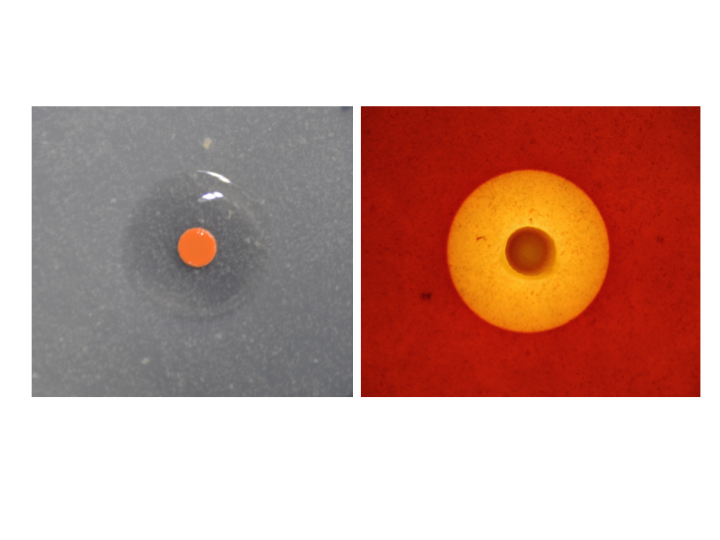

Supplement: Supplementary file 2 — Depression of an agar plate and clearance zone after staining with Lugol’s solution (0.2 % I2 plus 2 % KI). A drop of cell suspension of Halococcus sp. 197A was placed on the center of an agar plate and incubated at 37 °C for 2 weeks (TIFF 1521 kb) [file 792_2013_575_MOESM2_ESM.tiff]

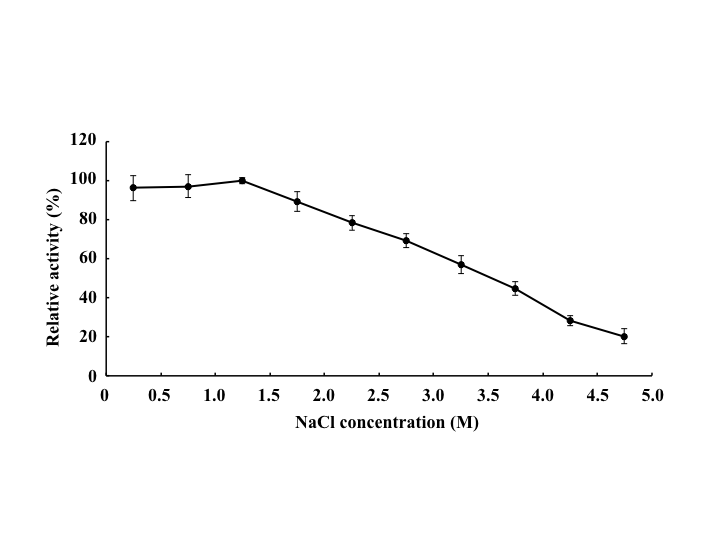

Supplement: Supplementary file 3 — The effect of NaCl on the enzyme activity and temperature stability of Thermostable β-Agarase from Microbulbifer thermotolerans. The enzyme activity was measured at NaCl concentrations ranging from 0.25 to 4.75 M using 5 mM Tris–HCl buffer (pH 7.0) (a) (TIFF 1521 kb) [file 792_2013_575_MOESM3_ESM.tiff]

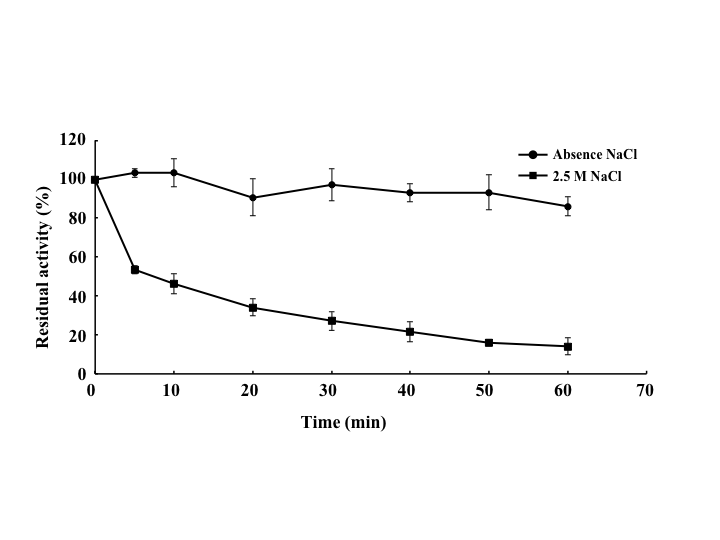

Supplement: Supplementary file 4 — For the thermal stability, the enzyme was incubated at 50 °C for 0–60 min in the absence (filled circles) or presence of 2.5 M NaCl (filled square), and remaining activities were measured at 50 °C for 15 min (TIFF 1521 kb) [file 792_2013_575_MOESM4_ESM.tiff]
